# Pre- and Postsynaptic Activation of GABA_B_ Receptors Modulates Principal Cell Excitation in the Piriform Cortex

**DOI:** 10.3389/fncel.2018.00028

**Published:** 2018-02-05

**Authors:** Leah B. Gerrard, Malinda L. S. Tantirigama, John M. Bekkers

**Affiliations:** Eccles Institute of Neuroscience, John Curtin School of Medical Research, The Australian National University, Canberra, ACT, Australia

**Keywords:** 2-photon, epilepsy, GABA_B_, modulation, piriform cortex

## Abstract

The piriform cortex (PC), like other cortical regions, normally operates in a state of dynamic equilibrium between excitation and inhibition. Here we examined the roles played by pre- and postsynaptic GABA_B_ receptors in maintaining this equilibrium in the PC. Using whole-cell recordings in brain slices from the anterior PC of mice, we found that synaptic activation of postsynaptic GABA_B_ receptors hyperpolarized the two major classes of layer 2 principal neurons and reduced the intrinsic electrical excitability of these neurons. Presynaptic GABA_B_ receptors are expressed on the terminals of associational (intracortical) glutamatergic axons in the PC. Heterosynaptic activation of these receptors reduced excitatory associational inputs onto principal cells. Presynaptic GABA_B_ receptors are also expressed on the axons of GABAergic interneurons in the PC, and blockade of these autoreceptors enhanced inhibitory inputs onto principal cells. Hence, presynaptic GABA_B_ autoreceptors produce disinhibition of principal cells. To study the functional consequences of GABA_B_ activation *in vivo*, we used 2-photon calcium imaging to simultaneously monitor the activity of ~200 layer 2 neurons. Superfusion of the GABA_B_ agonist baclofen reduced spontaneous random firing but also promoted synchronous epileptiform activity. These findings suggest that, while GABA_B_ activation can dampen excitability by engaging pre- and postsynaptic GABA_B_ heteroreceptors on glutamatergic neurons, it can also promote excitability by disinhibiting principal cells by activating presynaptic GABA_B_ autoreceptors on interneurons. Thus, depending on the dynamic balance of hetero- and autoinhibition, GABA_B_ receptors can function as variable modulators of circuit excitability in the PC.

## Introduction

The piriform cortex (PC) is a three-layered paleocortex that is thought to generate holistic representations of odors, using information about the chemical components of inhaled odors that is provided by the upstream olfactory bulb (OB; Gottfried, 2010; Wilson and Sullivan, 2011). Layer 2 of the PC contains high densities of two distinct populations of glutamatergic principal cells: semilunar (SL) cells in layer 2a that mostly receive afferent inputs from the OB; and superficial pyramidal (SP) cells in layer 2b that receive OB input and feedforward excitation from SL cells, as well as associational (intracortical) inputs from other SP cells (Neville and Haberly, 2004; Bekkers and Suzuki, 2013). SL and SP cells also receive feedforward inhibition from neurogliaform and horizontal cells, which are GABAergic interneurons that occupy the superficial layer 1a of the PC. Feedback inhibition is provided by a variety of interneurons in deeper layers, including bitufted, fast-spiking, chandelier, regular-spiking and deep neurogliaform cells (Suzuki and Bekkers, 2010a, b, 2012). These inhibitory circuits in the PC are of particular interest because of their modulatory roles in odor processing (Poo and Isaacson, 2011; Large et al., 2016).

GABA_B_ receptors are G-protein-coupled receptors of GABA which provide slow inhibitory control of synaptic transmission and neuronal excitability (Bettler et al., 2004). GABA_B_-mediated neurotransmission is important because it is critical for normal neural processing, as well as being linked to disorders like epilepsy, and because of the therapeutic use of the GABA_B_ receptor agonist baclofen in humans (Schuler et al., 2001; Schuele et al., 2005). GABA_B_ receptors are expressed postsynaptically in somatodendritic compartments, where their activation opens inwardly rectifying K^+^ channels (Newberry and Nicoll, 1985; Andrade et al., 1986). GABA_B_ receptors are also located presynaptically in the axon terminals of both excitatory neurons (heteroreceptors) and inhibitory interneurons (autoreceptors), where their activation inhibits Ca^2+^ influx via N or P/Q-type Ca^2+^ channels, leading to decreased neurotransmitter release (Harrison, 1990; Scholz and Miller, 1991; Bettler et al., 2004).

In the PC, it is known that postsynaptic GABA_B_ receptors give rise to a slow inhibitory postsynaptic potential in SP cells (Tseng and Haberly, 1988), but it is not known if a similar GABA_B_-mediated response is present in SL cells. On the presynaptic side, GABA_B_ heteroreceptors are known to be present on associational excitatory synapses on both SL and SP cells (Tang and Hasselmo, 1994; Franks and Isaacson, 2005; Suzuki and Bekkers, 2011), but the physiological conditions under which they might be activated are still unclear. GABA_B_ autoreceptors have also been reported on inhibitory presynaptic terminals in the PC (Kapur et al., 1997) but, again, it is unknown whether both SL and SP cells are the targets of these terminals and under what conditions these autoreceptors are active. Finally, questions remain about the significance of GABA_B_ receptor activation for odor processing *in vivo* (Poo and Isaacson, 2011; Riffell et al., 2014).

Here, we addressed these issues using whole-cell patch clamp recordings in brain slices and two-photon calcium imaging *in vivo*. We found that activation of postsynaptic GABA_B_ receptors hyperpolarize both SL and SP cells, reducing their excitability. We also found that GABA_B_ receptors located on glutamatergic or GABAergic terminals function as heteroreceptors or autoreceptors, respectively, to reduce EPSC or IPSC amplitudes in principal cells, with some differences between SL and SP cells. Finally, we found that GABA_B_ activation has dual effects on the PC circuit *in vivo*, on the one hand suppressing spontaneous activity in principal cells, and on the other hand promoting intermittent epileptiform activity in the same cells. Hence, GABA_B_ receptors can function as variable modulators of the piriform circuit, with the outcome depending upon the balance of inhibitory and disinhibitory mechanisms they engage.

## Materials and Methods

### Animals

All animal housing, breeding and surgical procedures were approved by the Animal Experimentation Ethics Committee of the Australian National University and conform to the guidelines of the National Health and Medical Research Council of Australia. Experiments used heterozygous GAD67-GFP (∆neo) mice of either sex bred on a C57BL6/J background.

### Slice Electrophysiology

Experiments used acute brain slices (300 μm thick) prepared from 16-day to 26-day-old mice using standard methods as previously described (Suzuki and Bekkers, 2006). Briefly, mice were deeply sedated with isoflurane (2.5% in oxygen), decapitated, and the brain immediately removed and submerged in ice-cold cutting solution (in mM: 125 NaCl, 3 KCl, 25 NaHCO_3_, 1.25 NaH_2_PO_4_, 0.5 CaCl_2_, 6 MgCl_2_, 10 glucose, 1.5 (+)-sodium L-ascorbate, 2.25 sodium pyruvate and saturated with 95% O_2_/5% CO_2_). Coronal slices containing the anterior PC were cut using a Vibroslice (Campden Instruments) and incubated in artificial cerebrospinal fluid (ACSF, in mM: 125 NaCl, 3 KCl, 25 NaHCO_3_, 1.25 NaH_2_PO_4_, 2 CaCl_2_, l MgCl_2_, 25 glucose, bubbled with 95% O_2_/5% CO_2_) at 35 °C for 45 min, then at room temperature until required.

For electrophysiological recordings, slices were transferred to a recording chamber and continuously superfused (2–3 ml/min) with warmed (31 ± 1.5°C) ACSF. Infrared-differential interference contrast microscopy (Olympus BX50WI) was used to make visualized whole-cell patch clamp recordings from layer 2 principal neurons in the PC. The two main classes of these neurons, SL and SP cells, were identified by their characteristic electrical properties and laminar location (Suzuki and Bekkers, 2011). Recording electrodes were glass pipettes with a resistance of 5–7 MΩ when filled with (in mM) 135 KMeSO_4_, 7 NaCl, 10 HEPES, 0.1 EGTA, 2 MgCl_2_, l Na_2_ATP, 0.3 Na_3_GTP, 10 sorbitol adjusted to pH 7.2 with KOH. In experiments in which IPSCs were recorded, 135 CsMeSO_4_ replaced KMeSO_4_ to block K^+^ channels. Data were obtained using a Multiclamp 700A amplifier (Molecular Devices), filtered at 10 kHz and digitized at 20 or 50 kHz by an ITC-18 interface (Instrutech/HEKA) under the control of Axograph (Axograph Scientific). Unless otherwise stated, current clamp recordings were done at the resting potential of the neuron (SP: −80.3 ± 6.7 mV, *n* = 90; SL: −61.3 ± 0.7 mV, *n* = 43), and the voltage clamp recordings were done with the soma holding potential at −70 mV. Pipette capacitance was neutralized and the series resistance compensated in current clamp with the bridge balance circuit. No liquid junction potential correction was applied.

Focal extracellular synaptic stimulation of the PC was done using an isolated stimulator (Digitimer DS2) that delivered 100 μs long constant-voltage pulses with an adjustable amplitude. Stimuli were delivered at 20 s intervals. The stimulating electrode was constructed from a low resistance patch electrode (tip diameter 5–10 μm) filled with ACSF and coated with electrically-conductive paint. The stimulus current was passed between the filling solution and a wire connected to the paint; hence, this functioned as a concentric bipolar stimulating electrode (Bekkers and Clements, 1999). The tip of the stimulating electrode was placed in different layers (layer 1a, 1b, 2 or 3) by reference to established neuroanatomical landmarks (Suzuki and Bekkers, 2011) in order to achieve layer-specific stimulation. The tip was always at least 65 μm away from the recorded soma to avoid direct stimulation of the neuron.

ACSF was supplemented with picrotoxin (100 μM) to block GABA_A_ receptors, plus 2,3,4-tetrahydro-7-nitro-2,3-dioxoquinoxaline-6-carbonitrile disodium (CNQX; 10 μM) and D-2-amino-5-phosphonopentanoic acid (D-APV; 50 μM) to block glutamate receptors when recording postsynaptic GABA_B_ responses. EPSCs were recorded in the absence of CNQX and D-APV, and IPSCs were recorded in the absence of picrotoxin. Prior to recording IPSCs, the soma was clamped at 0 mV in voltage clamp to increase the driving force for Cl^−^ ions.

### *In Vivo* Two-Photon Calcium Imaging and LFP Recording

Surgical procedures for exposing the anterior PC and conducting two-photon microscopy were performed as described previously (Tantirigama et al., 2017). Briefly, mice (50–70 days-old) were anesthetized using a cocktail of chlorprothixene (5 mg/kg), urethane (0.7 g/kg) and atropine (0.2 mg/kg) delivered subcutaneously. Skin incisions were treated with a local anesthetic (prilocaine, 0.2 mg/kg). The depth of anesthesia was monitored throughout the experiment; when necessary, a top-up dose of urethane (10%–30% of the initial dose) was given. Body temperature was maintained at 36–37°C using a heating blanket. A craniotomy (~1.5 × 1.5 mm) was opened over the region where the middle cerebral artery and the dorsal aspect of the lateral olfactory tract (LOT) intersect, and the dura was removed. Using dental cement (Paladur, Heraeus) a headpost was attached to the skull for head-fixation and then a water-tight chamber was constructed around the surgical site to accommodate the water-immersion objective. The chamber was filled with Ringer’s solution containing (mM) 135 NaCl, 5.4 KCl, 1.8 CaCl_2_, 1 MgCl_2_, 5 HEPES at pH 7.4. The calcium indicator dye Cal-520 AM (1 mM; AAT Bioquest) was pressure-injected into the PC at a depth of 200–300 μm with a micropipette. Sulforhodamine (SR) 101 (50 μM) was added to the Cal-520 solution to label astrocytes. Calcium imaging was performed at least 30 min after dye loading.

A piece of No. 0 glass coverslip was cut to fit over the craniotomy and glued in place. Imaging was done using a Thorlabs two-photon microscope with a 16× water immersion objective (Nikon, 0.8 NA), resonant-galvanometer scanners, and a Ti:Sapphire laser (Chameleon Ultra, Coherent) running at 810 nm. The x-y image scanning plane was either a 300 × 300 μm (512 × 512 pixels) or a 300 × 150 μm (512 × 256 pixels) frame, and time-series movies were captured at 30 or 60 frames per second, respectively. SL and SP cells were distinguished by their laminar location and depth from the pial surface (SL: layer 1b/2a border, 160–210 μm deep; SP: layer 2b/3 border, 250–300 μm deep).

Local field potential (LFP) recordings were done using patch pipettes (1–4 MΩ) filled with Ringer solution positioned in layer 2 of PC at a 30° angle and a depth of 200–300 μm. Recordings were filtered at 800 Hz and digitized at 5 kHz by using a Multiclamp 200B amplifier (Molecular Devices) and an ITC-18 interface. Respiration was monitored using a piezoelectric strap around the chest.

### Data Analysis

**Figure 1:**
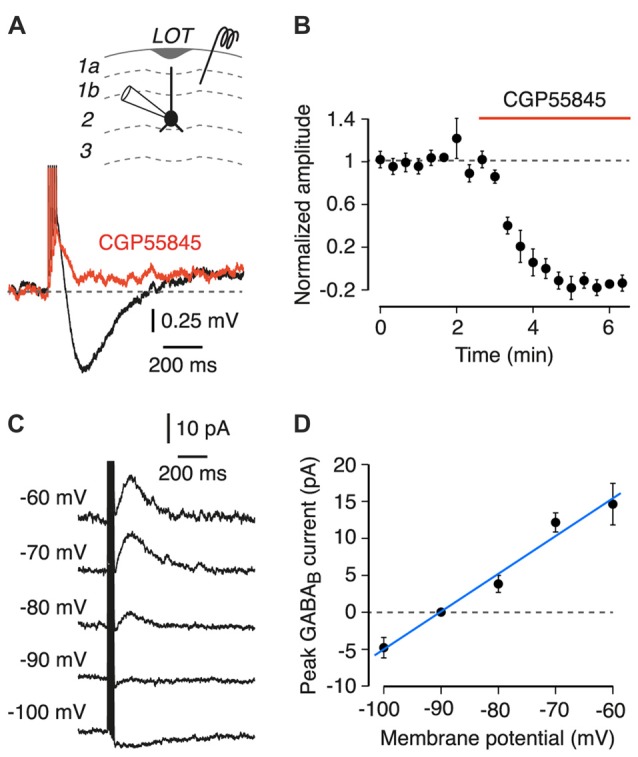
Properties of a postsynaptic GABA_B_ receptor-mediated response in semilunar (SL) and superficial pyramidal (SP) cells in layer 2 of the piriform cortex (PC). Inset at top shows a schematic of the experiment configuration. LOT, lateral olfactory tract. **(A)** Layer 1 stimulation (five pulses at 100 Hz) evokes a slow hyperpolarization (black trace) that is blocked by 5 μM CGP55845 (red; each trace is an average of 10 episodes; SP cell shown). **(B)** Mean peak amplitude of the postsynaptic GABA_B_ receptor response, normalized to the mean amplitude during the baseline period, plotted vs. time (*n* = 7 SL and SP cells). Normalized amplitude decreases after bath application of CGP55845 (red bar). Error bars show ± SEM. **(C)** Typical voltage-clamp experiment for determining the reversal potential of the GABA_B_-mediated postsynaptic current. Each trace is an average of four episodes, each at the indicated holding potential. Stimulus as in **(A)**. Data are from an SP cell. **(D)** Mean current-voltage plot for the cell in **(C)**. Blue line is a linear fit, giving an estimated reversal potential of −91 mV in this example. Error bars show ± SD.

**Figure 2:**
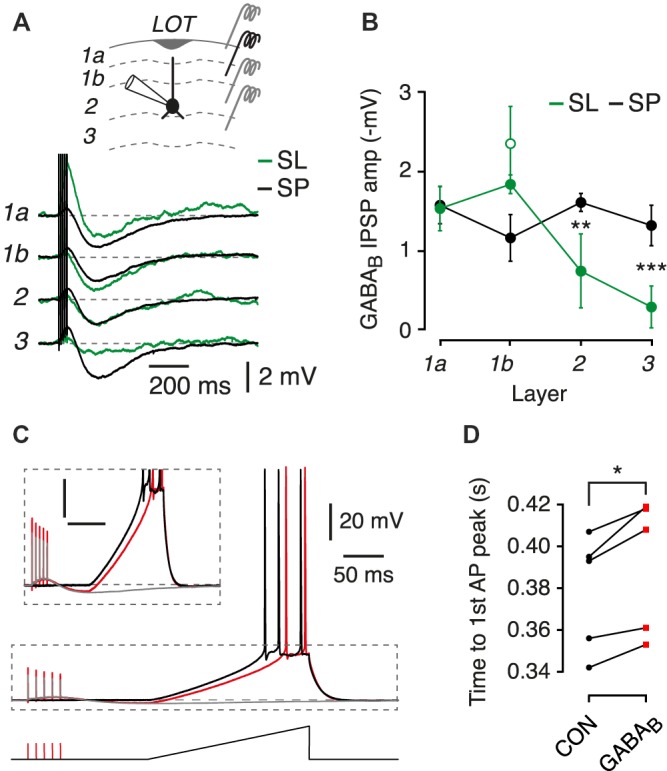
Postsynaptic GABA_B_ receptors suppress the excitability of SL and SP cells. **(A)** Typical GABA_B_ receptor-mediated postsynaptic potentials in an SL cell (green) and an SP cell (black) resulting from 5-pulse 100 Hz stimuli applied in the layer indicated at left. Each trace is an average of four episodes. Inset shows a schematic of the experiment configuration, with the different stimulator locations indicated. **(B)** Mean peak postsynaptic GABA_B_ IPSP plotted against layer number for SL cells (green; *n* = 3) and SP cells (black; *n* = 5). Green open symbol shows the mean bracketing response of SL cells to layer 1b stimulation at the end of the experiment, confirming absence of run-down. Error bars show ± SEM. **(C)** Typical result from an experiment in which a depolarizing current ramp was applied in the absence (black trace) or presence (red trace) of a preceding 5-pulse 100 Hz stimulus train to elicit a postsynaptic GABA_B_ receptor response. Gray trace shows the GABA_B_ receptor response without the current ramp. Data are from an SP cell. Region in the dashed box is shown expanded in the inset. Scale bars for inset: 10 mV, 100 ms. **(D)** Summary data for experiments like in **(C)** showing that prior activation of postsynaptic GABA_B_ receptors increases the latency to the first action potential (AP) fired in response to the current ramp. CON, control. Combined data from *n* = 5 SL and SP cells. *ns*, not significant; *p < 0.05; **p < 0.01; ***p < 0.001.

Electrophysiology analysis was done using Axograph. Latency to first AP was defined as the time from the beginning of current ramp to the peak of the first AP. The synaptically-evoked responses shown in the figures and used for analysis were averages of 4–10 individual episodes (see figure legends). The peak of the postsynaptic GABA_B_ receptor-mediated response was found by averaging the voltage or current during a window of length 20 ms (Figure 1) or 50 ms (Figures 2A,B), centered around the peak synaptic response relative to baseline. The baseline was adjusted over a 50 ms window preceding the first stimulus. Analysis of EPSCs and IPSCs was similar, except that the peak was measured over a 1–3 ms window and the baseline was adjusted over a 5 or 20 ms window, respectively. The paired-pulse ratio (PPR) of synaptic currents was calculated as the amplitude of the second or third current divided by that of the first.

Fluorescence changes in calcium imaging data were analyzed with ImageJ^1^ and custom MATLAB code (MathWorks). Images in each time-series movie were registered using the ImageJ plugin Turboreg, and regions of interest (ROIs) were drawn manually for each individual soma (excluding astrocytes labeled with SR 101) in the average registered image. The raw fluorescence intensity of each ROI was calculated and expressed as ∆F/F_0_ = (F − F_0_)/F_0_, where F_0_ is the median of the lower 80% of values. Transients in the ∆F/F_0_ trace were detected using a sliding template and converted to spike rate as described previously (Tantirigama et al., 2017). All analysis was done using unfiltered traces, but a moving-average three-point filter was used to smooth the traces shown in the figures.

### Statistical Analysis

Statistical analysis was done using Prism 6.0 (GraphPad) and R^2^. Comparisons used linear mixed effects regression models, analysis of variance (ANOVA) or the 2-tailed *t*-test, where appropriate and as indicated. Linear models were calculated using the lmer package in R by testing the relationship between variables, and included both fixed effects (such as amplitude or drug) and random effects (such as inter-neuron variation), avoiding pseudo-replication. A *t*-test on the coefficient of fitted linear regression was used to assess whether the coefficient was significantly different from zero. Data from SL and SP cells were combined if statistical analysis indicated that there were no cell-type dependent effects, with the exception of Figure 4, in which the analysis was run separately for SL and SP cells.

**Figure 3:**
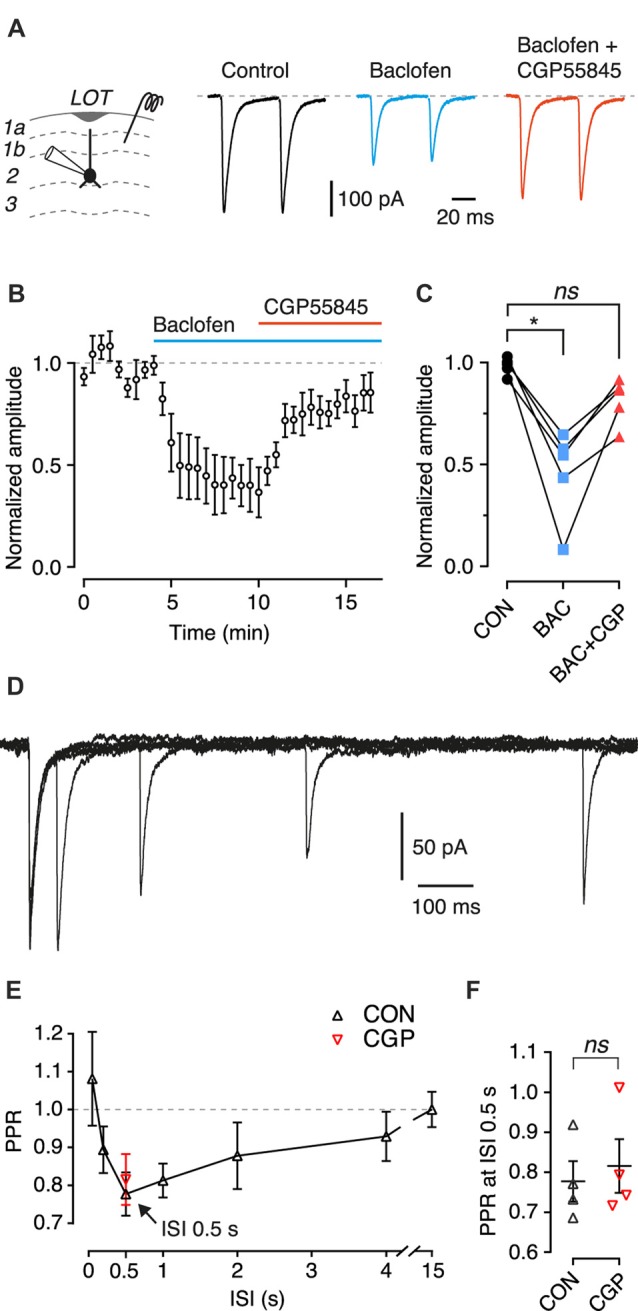
Activation of presynaptic GABA_B_ heteroreceptors inhibits EPSCs from associational inputs onto SP cells, but these receptors are not involved in paired-pulse depression. **(A)** Typical EPSCs recorded in an SP cell in response to paired-pulse stimulation (50 ms interstimulus interval, ISI) of layer 1b during control (black), in the presence of baclofen (100 μM; blue) and baclofen + CGP55845 (5 μM; red). Each trace is an average of 10 episodes. Stimulus artifacts have been blanked. **(B)** Mean EPSC amplitude, normalized to the mean amplitude during the control period, plotted vs. time. Baclofen (bath applied during the period shown by the blue bar) decreased the normalized EPSC amplitude, and addition of CGP55845 (application shown by the red bar) reversed this inhibition. Error bars are ± SEM. Data from *n* = 5 SP cells. **(C)** Summary data for **(B)** showing the normalized EPSC amplitude averaged over the control period (CON, 100–190 s), the period in baclofen (BAC, 400–490 s) and the period in baclofen + CGP55845 (BAC+CGP, 800–890 s). Symbols show means for individual cells (*n* = 5 SP cells). **(D)** Typical data from an EPSC paired-pulse experiment with an SP cell and ISI 50, 200, 500 and 1000 ms. Each trace is an average of five episodes. Stimulus artifacts have been blanked. **(E)** Mean paired-pulse ratio (PPR) plotted vs. ISI (*n* = 4 SP cells). Arrow indicates the ISI with the smallest PPR (0.5 s). Black symbols show the mean PPR measured under control conditions; red symbol shows the mean PPR measured at 0.5 s ISI in the same cells in the presence of 5 μM CGP55845 (*n* = 4 SP cells). Error bars show ± SEM. **(F)** Summary data for **(E)** showing PPR at 0.5 s ISI during control (CON) and in the presence of CGP55845 (CGP). Symbols show the mean for individual cells (*n* = 4 SP cells). Error bars show ± SEM. *ns*, not significant; *p < 0.05.

**Figure 4:**
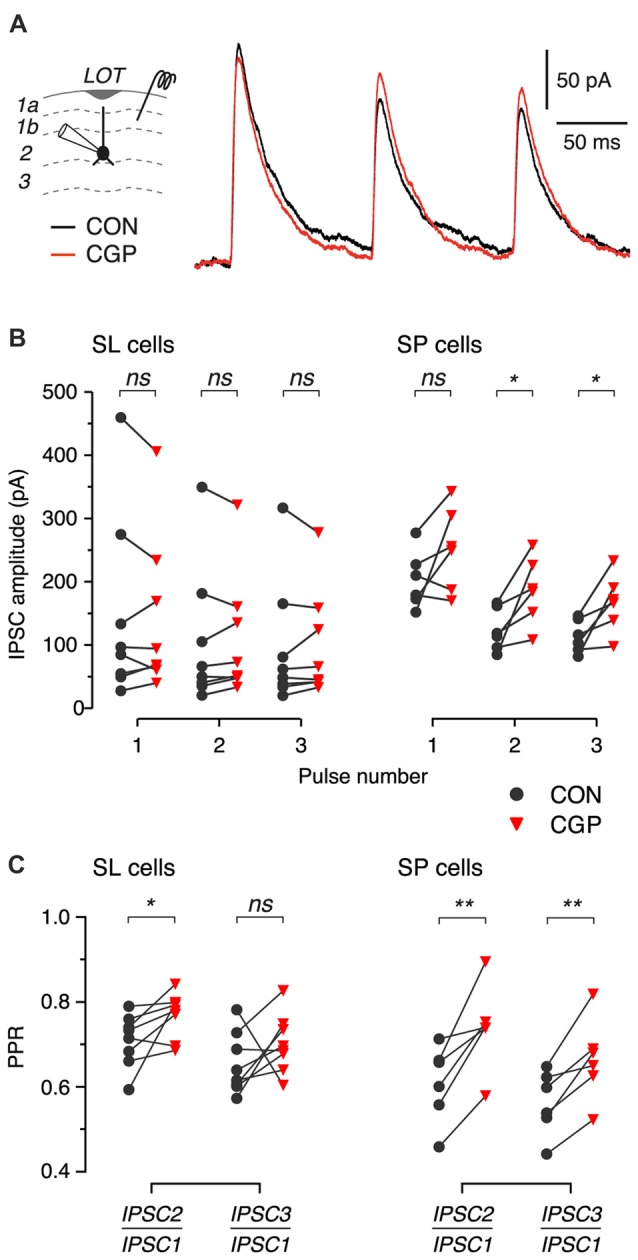
Blockade of presynaptic GABA_B_ autoreceptors increases the amplitude and PPR of IPSCs in SP cells. **(A)** Typical IPSCs recorded in response to 3-pulse train stimulation (200 ms ISI) of layer 1b during control (CON, black) and after bath application of 5 μM CGP55845 (CGP, red). Data are from an SP cell. Each trace is an average of 8 episodes. Stimulus artifacts have been blanked. **(B,C)** Summary of IPSC amplitude **(B)** and PPR **(C)** before (black) and after (red) application of CGP55845 for *n* = 8 SL cells and *n* = 6 SP cells. *ns*, not significant; *p < 0.05; **p < 0.01.

## Results

### Postsynaptic GABA_B_ IPSPs and IPSCs Can Be Elicited in SL and SP Cells

We searched for postsynaptic GABA_B_ receptor responses by applying a 5-pulse 100 Hz extracellular electrical stimulus to layer 1a while recording from an identified SL or SP cell. The bath solution contained 10 μM CNQX, 25 μM D-APV and 100 μM picrotoxin to block AMPA, NMDA and GABA_A_ receptors, respectively. Recordings in current clamp mode revealed a fast depolarization followed by a slow hyperpolarization which peaked at 185 ± 0.01 ms and had an average amplitude of 1.34 ± 0.08 mV (*n* = 7, combined data from SL and SP cells, no significant difference between cell types, *p* = 0.6, linear mixed effects model; Figure 1A, black trace). This hyperpolarization was blocked by the GABA_B_ receptor antagonist CGP55845 (5 μM; Figure 1A, red trace). The block was quantified by plotting the mean normalized amplitude of the hyperpolarization vs. time (Figure 1B). CGP55845 significantly reduced the normalized amplitude to −0.02 ± 0.06 (*n* = 7, *p* < 0.001, linear mixed effects model), suggesting that the hyperpolarization was due to GABA_B_ receptor activation. The small, fast depolarization preceding the GABA_B_ response was most likely due to incompletely blocked ionotropic glutamate receptors.

To confirm the identity of the slow hyperpolarization, the reversal potential of the underlying current was measured in voltage clamp mode (Figure 1C, example traces at a range of holding potentials, measured for an SP cell; Figure 1D, current-voltage plot for the same cell). Current-voltage plots gave an average reversal potential of −91.9 ± 1.4 mV and an average conductance of 0.38 ± 0.06 nS (*n* = 11, combined data from SL and SP cells). The reversal potential is similar to the calculated Nernst potential for K^+^ for our solutions (−100 mV) and is consistent with the GABA_B_ response being mediated by a K^+^-selective channel (Bettler et al., 2004).

### Layer-Specific Differences in Postsynaptic GABA_B_ IPSPs in SL and SP Cells

We next asked whether the size of the postsynaptic GABA_B_ response depended on the layer to which stimulation was applied, and whether such differences might be cell-type dependent. We hypothesized that, since SL cells lack basal dendrites and SP cells do not (Suzuki and Bekkers, 2011), SL cells would show smaller GABA_B_ synaptic responses when extracellular stimulation was applied to deeper layers (layers 2 and 3).

SP cells showed a consistent GABA_B_ response which did not differ between layers (Figures 2A,B, black traces and symbols; *n* = 5, *p* > 0.05, ANOVA). In contrast, SL cells showed a significantly decreased response in deeper layers compared to that in SP cells (layer 2: *p* = 0.007; layer 3: *p* = 0.0006, ANOVA; SP, *n* = 5; SL, *n* = 3). Hence, SL and SP cells receive similar GABA_B_ input in layer 1, but SP cells receive stronger GABA_B_ input in deeper layers, consistent with the known differences in dendritic morphology (Suzuki and Bekkers, 2011; Choy et al., 2017).

### Postsynaptic GABA_B_ IPSPs Reduce Excitability of Principal Cells

The physiological consequence of activating postsynaptic GABA_B_ receptors in SL and SP cells was assessed using a protocol in which a constant current ramp was used to initiate action potentials either with or without a preceding GABA_B_ response (Figure 2C). The latency to the first action potential was significantly delayed in both SL and SP cells when a GABA_B_ IPSP was present (Figure 2D; *p* = 0.014, paired *t*-test, *n* = 5, combined data from SL and SP cells). Moreover, the number of action potentials was significantly reduced following GABA_B_ receptor activation (control, 2.9 ± 0.2; GABA_B_, 2.3 ± 0.2; *p* < 0.001, paired *t*-test, *n* = 5, combined data from SL and SP cells). Thus, by several measures the intrinsic excitability of layer 2 principal cells is reduced by the prior occurrence of a GABA_B_ IPSP.

### Presynaptic GABA_B_ Heteroreceptors Can Suppress Associational EPSCs in SP Cells

We next turned to a study of presynaptic GABA_B_ receptors. EPSCs were recorded under voltage clamp in SP cells while applying paired extracellular electrical stimuli (50 ms between stimulus pairs) to associational fibers in layer 1b (Figure 3A). Bath perfusion of the GABA_B_ receptor agonist baclofen (100 μM) caused a large decrease in the EPSC amplitude (Figures 3B,C; *p* < 0.05; repeated measures ANOVA and Dunnett’s *post hoc* test, *n* = 5). This inhibition was partly reversed by CGP55845 (Figures 3B,C). The PPR of the EPSC amplitudes was significantly increased in the presence of baclofen (control: 0.96 ± 0.05; baclofen: 1.16 ± 0.12; *p* = 0.005, 2-tailed *t*-test, *n* = 5 cells), indicating that the EPSC suppression has a presynaptic locus of expression. These results are consistent with previous reports (Tang and Hasselmo, 1994; Franks and Isaacson, 2005; Suzuki and Bekkers, 2011).

### Presynaptic GABA_B_ Heteroreceptors Are Not Involved in Paired-Pulse Depression of Associational EPSCs in SP Cells

Having verified that exogenously applied baclofen can presynaptically inhibit associational EPSCs in SP cells, we next wished to examine the effects of presynaptic GABA_B_ receptor activation in a more physiological setting, without the use of baclofen. To do this, we designed a paired-pulse protocol in which the interstimulus interval (ISI) was randomly varied over a range of durations between 50 ms and 15 s (Figure 3D). Under control conditions a short ISI (50 ms) produced little paired-pulse facilitation or depression, consistent with the result in the previous section and earlier work (Suzuki and Bekkers, 2006). However, slightly longer ISIs (200 ms and 500 ms) produced clear paired-pulse depression, with the response fully recovered at 15 s (Figure 3E).

We hypothesized that the depression at intermediate ISIs was due to the activation of GABA_B_ heteroreceptors: GABA released from inhibitory terminals during the first stimulus might diffuse to nearby excitatory terminals and presynaptically inhibit the EPSC response to the second stimulus. The slow timecourse of the depression might reflect the time required for diffusion and presynaptic suppression of glutamate release (Isaacson et al., 1993). This idea was tested by repeating the experiment in the presence of the GABA_B_ receptor antagonist CGP55845 (5 μM), which was predicted to reverse the depression at 500 ms. Contrary to expectations, no significant difference was found before and after adding CGP55845 (Figure 3F; control: 0.81 ± 0.03; CGP: 0.76 ± 0.02; *p* = 0.69, paired *t*-test, *n* = 4). This result suggests that GABA_B_ receptors do not mediate the paired-pulse depression observed at 500 ms with this protocol.

### Presynaptic GABA_B_ Autoreceptors Increase Short-Term Depression of IPSCs in SL and SP Cells

Given that we were unable to reveal GABA_B_ heteroreceptors on glutamate-releasing terminals in response to paired-pulse electrical stimulation in slices, we wondered if similar or stronger stimuli might unmask GABA_B_ autoreceptors on GABA-releasing terminals. We addressed this question by recording IPSCs in identified SL and SP cells while applying brief trains of extracellular stimuli to associational layers (three pulses with 200 ms ISIs; Figure 4A; extension of a protocol used by Kapur et al., 1997). IPSCs were isolated by pharmacologically blocking ionotropic glutamate receptors and by voltage-clamping the neurons at 0 mV to increase the electrical driving force for Cl^−^ (“Materials and Methods” section). The trains of IPSCs were recorded before and after bath perfusion of CGP55845 (5 μM) to block GABA_B_ receptors.

We expected to see GABA_B_ receptor-dependent effects on the later IPSCs in the train, due to activation of GABA_B_ autoreceptors on the presynaptic GABAergic terminals. We did find such effects, which were more prominent in SP cells (Figure 4B). While the amplitude of the first IPSC in the train was not significantly affected by GABA_B_ receptor blockade in either cell type (SL: 147.9 ± 52.2 pA in control, 142.5 ± 44.4 pA in CGP, *p* = 0.64, linear model, *n* = 8; SP: 203.3 ± 18.5 pA in control, 251.9 ± 27.1 in CGP, *p* = 0.12, linear model, *n* = 6), the amplitudes of the second and third IPSCs were significantly increased by CGP55845 in SP cells but not in SL cells (e.g., second IPSC; SL: 106.3 ± 39.3 pA in control, 109.1 ± 34.4 pA in CGP, *p* = 0.70, linear model, *n* = 8; SP: 123.9 ± 13.8 pA in control, 186.6 ± 21.6 in CGP, *p* = 0.025, linear model, *n* = 6). Furthermore, the presynaptic locus of this effect was confirmed by the significant increase in PPR in SP cells (Figure 4C, right). There was also a small but significant increase in this ratio for the first and second IPSCs in SL cells (Figure 4C, left). These findings suggest that synaptically released GABA can activate presynaptic GABA_B_ autoreceptors on inhibitory terminals to suppress later IPSCs in a train, but the effect appears to be stronger at inhibitory synapses onto SP cells.

### Activation of GABA_B_ Receptors *in Vivo* Both Suppresses and Enhances Network Activity in SL and SP Cells

Our results so far have indicated that activation of GABA_B_ receptors can have both inhibitory and excitatory effects on neural circuits in the PC. On the one hand, activation of postsynaptic GABA_B_ receptors inhibits firing in SL and SP cells (Figure 2), and presynaptic GABA_B_ heteroreceptors are poised to inhibit glutamate release (Figure 3). Both of these effects are inhibitory. On the other hand, activation of presynaptic GABA_B_ autoreceptors can reduce GABA release onto SP cells (Figure 4), thereby disinhibiting those cells and having an overall excitatory effect on the circuit. We wondered if both inhibitory and excitatory effects of GABA_B_ receptor activation could be observed in the intact PC *in vivo*.

**Figure 5:**
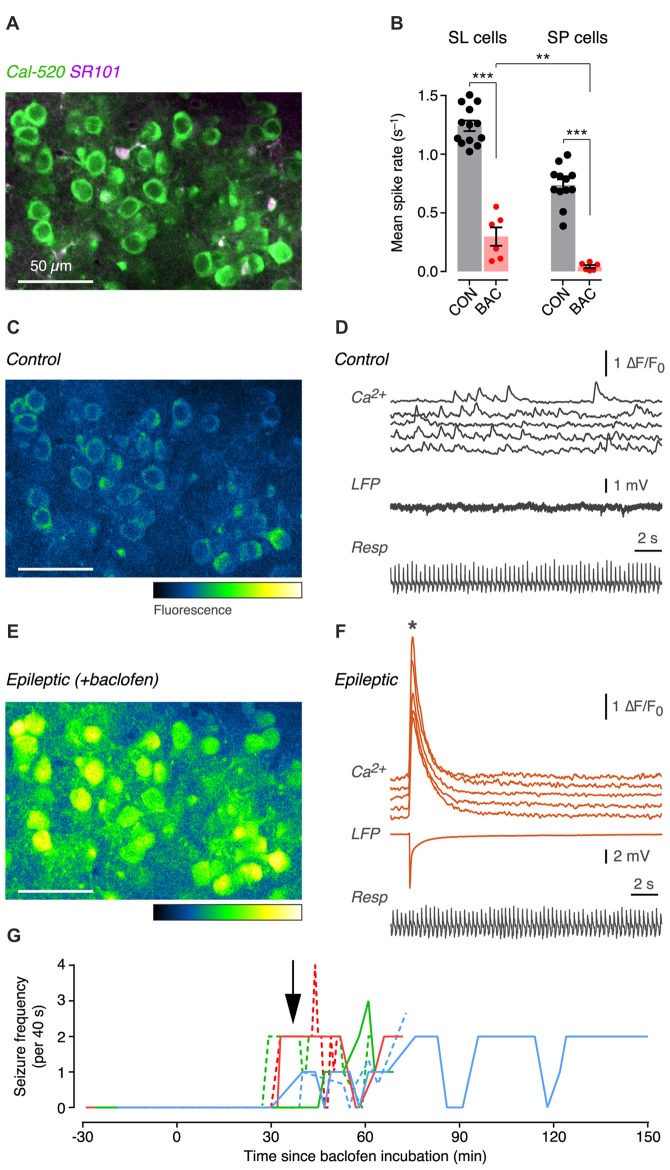
GABA_B_ receptor activation both suppresses and enhances the excitability of SL and SP cells in the PC *in vivo*. **(A)** Two-photon composite image of SP cell somas labeled with the calcium indicator, Cal-520. Sulforhodamine (SR101) labels astrocytes. **(B)** Mean spontaneous spike rates during control (CON, black) and after superfusion of 500 μM baclofen (BAC, red), measured for SL cells (*left*) and SP cells (*right*). Each point is the averaged firing rate for all cells (*n* ~ 60–230 cells) in a field of view in a single experiment (SL: CON, *n* = 13 mice; BAC, *n* = 6; SP: CON, *n* = 12; BAC, *n* = 6). Shaded bars show the mean; error bars show ± SEM. **(C)** Example pseudocolor image of fluorescence intensity in the field shown in **(A)** under control conditions. Pseudocolor fluorescence scale shows low to high intensity. **(D)** Simultaneously recorded ∆F/F_0_ traces from five cells in the field in **(C)** showing spontaneous activity (*top*), the local field potential (LFP) recording (*middle*) and the respiration of the animal (*bottom*) under control conditions. **(E)** Same as in **(C)** but 36 min after baclofen was added to the superfusate. The image was taken at the time of the epileptic event indicated by the asterisk in **(F)**. **(F)** Same as in **(D)**, but after 36 min of baclofen. Note the large synchronous events seen in both the ∆F/F_0_ traces and the LFP trace. **(G)** Timecourse plot of the number of epileptic events observed in each 40 s-long movie of calcium fluorescence vs. the time since baclofen was first applied to the cortical surface. Each colored line shows the timecourse plot for each of *n* = 6 mice. Arrow indicates the mean latency to onset of the first seizure event, averaging across all mice. **p < 0.01; ***p < 0.001.

To address this question, we performed *in vivo* two-photon calcium imaging of identified SL and SP cells in the anterior PC of anesthetized mice before and after superfusing the cortical surface with the GABA_B_ receptor agonist, baclofen (500 μM). SL and SP cells, loaded with the calcium indicator Cal-520, were distinguished from each other by their cortical depth and from astrocytes by their exclusion of the glial marker SR101 (e.g., Figure 5A, showing SP cells). Under control conditions in the absence of baclofen, individual SL and SP somas exhibited random spontaneous calcium transients (Figures 5C,D, upper traces), each of which corresponds to the firing of ≥2 action potentials (Tantirigama et al., 2017). As we have shown previously, this random firing is driven by spontaneous bottom-up input from the OB and is not synchronized to respiration (Figure 5D, bottom trace; Tantirigama et al., 2017).

Addition of baclofen (500 μM) to the Ringer’s solution bathing the exposed surface of the cortex quickly (<15 min) suppressed the mean spontaneous firing rate in both SL cells and SP cells (Figure 5B; SL: control, 1.24 ± 0.04 s^−1^, *n* = 13 mice; baclofen, 0.30 ± 0.08 s^−1^, *p* < 0.001, unpaired *t*-test, *n* = 6; SP: control, 0.74 ± 0.05 s^−1^, *n* = 12 mice; baclofen, 0.04 ± 0.01 s^−1^, *p* < 0.001, unpaired *t*-test, *n* = 6). The baclofen-suppressed spontaneous firing rate was significantly smaller in SP cells compared to SL cells (Figure 5B; *p* < 0.01, unpaired *t*-test). Thus, the initial effect of activating GABA_B_ receptors with baclofen was to inhibit the overall excitability of the anterior PC by suppressing spontaneous spiking activity.

With more prolonged exposure to baclofen (>29 min), large synchronized calcium transients started to emerge within both the SL cell and SP cell populations (Figures 5E–G, upper traces; asterisk indicates the time point shown in Figure 5E). A local field potential (LFP) electrode inserted in the PC also registered a large electrical event corresponding to the synchronized calcium transient (Figure 5F, middle trace; compare with corresponding trace in Figure 5D before the emergence of synchronized activity). A timecourse plot showing the onset of synchronized activity after baclofen application (Figure 5G) indicates that epileptiform activity appeared after 37 ± 6.5 min (mean ± SD, *n* = 6 mice). This kind of hyperexcitability was never seen in control animals during long experiments (>120 min) in the absence of baclofen (*n* > 50 mice). Hence, extended exposure to baclofen causes stronger excitatory effects of GABA_B_ receptor activation *in vivo*.

## Discussion

Modulation of neural activity by pre- and postsynaptic GABA_B_ receptors has been reported in many brain areas (Bettler et al., 2004), including the PC (Neville and Haberly, 2004). In this article we have extended earlier findings by studying the effects of GABA_B_ receptor activation on identified subtypes of PC principal neurons (SL and SP cells) and by exploring some of the consequences of GABA_B_ receptor activation *in vivo*.

We found that both SL and SP cells express postsynaptic GABA_B_ receptors that generate a slow IPSP in response to synaptic stimulation (Figures 2A,B). In both cell types, the effect of this IPSP was to subtly suppress the probability of firing action potentials in the postsynaptic neuron (Figures 2C,D). We also confirmed earlier findings that pharmacological activation of presynaptic GABA_B_ receptors on glutamatergic association-fiber terminals depressed EPSCs recorded in SL and SP cells (Figures 3A–C). However, we found no evidence for (quasi-) physiological activation of these GABA_B_ receptors when using a paired-pulse stimulus protocol to try to produce heterosynaptic activation via spillover of GABA at least when measuring at ISI = 500 ms Figures 3D–F). On the other hand, we did find evidence for a presynaptic GABA_B_ autoreceptor-mediated modulation of IPSCs when using a triple-pulse stimulus protocol (Figure 4). This effect appeared to be more prominent in SP cells (Figures 4B,C). Finally, we found that local *in vivo* application of the GABA_B_ agonist, baclofen, had a bimodal effect: initially, baclofen inhibited spontaneous spiking activity in SL and SP cells, and then later it caused synchronous epileptiform excitation (Figure 5). These *in vivo* observations may suggest that different GABA_B_-mediated effects—first inhibitory, later excitatory—may be progressively engaged as the agonist diffuses deeper into the intact PC (see below). Overall, our findings highlight that GABA_B_ receptors can function as potent modulators of the balance between excitation and inhibition in the piriform circuit.

It is important to maintain a distinction between SL and SP cells because we and others have shown that these cell types are morphologically, genetically and functionally distinctive, and so are likely to play different roles in the operation of the PC (Haberly, 1983; Suzuki and Bekkers, 2006, 2011; Wiegand et al., 2011; Carceller et al., 2016; Choy et al., 2017; Tantirigama et al., 2017). Previous work on GABA_B_ receptors in the PC, however, has often neglected to maintain this distinction between subtypes of layer 2 principal cells. For example, an early report described a slow IPSC in generic layer 2 pyramidal cells, probably mediated by postsynaptic GABA_B_ receptors (Tseng and Haberly, 1988). Here we found that SL and SP cells receive similar GABA_B_ IPSCs when the stimulator is in layer 1, but the GABA_B_ IPSC amplitude in SL cells falls off dramatically when stimulating in deeper layers (Figures 2A,B). This finding is consistent with the lack of basal dendrites in SL cells (Suzuki and Bekkers, 2011; Choy et al., 2017). Interestingly, our estimate of the conductance of GABA_B_ IPSCs with layer 1 stimulation (about 0.4 nS) is smaller than the conductance found in hippocampal pyramidal cells under similar stimulation conditions (0.9–1.5 nS; Otis et al., 1993; Ling and Benardo, 1994), suggesting that this form of slow inhibition is less significant for the PC.

Presynaptic GABA_B_ receptors on excitatory terminals have previously been shown to be restricted to associational (intracortical) inputs onto generic layer 2 principal cells; they are not found on afferent inputs from the OB (Tang and Hasselmo, 1994; Franks and Isaacson, 2005). More recently we confirmed that SL and SP cells both follow the same rule (Suzuki and Bekkers, 2011). However, all of these studies used exogenous baclofen to activate the presynaptic GABA_B_ receptors (as in Figures 3A–C). Our observation of a slow paired-pulse depression of EPSCs at associational inputs onto SP cells (Figures 3D,E) lead us to wonder if this depression could be explained by spillover of endogenous GABA from nearby inhibitory terminals (as in the hippocampus; Isaacson et al., 1993). Surprisingly, blockade of GABA_B_ receptors had no effect when assayed at 500 ms ISI (Figures 3E,F). It is possible that the depression is due to presynaptic metabotropic glutamate receptors (mGluRs) acting as autoreceptors (Baskys and Malenka, 1991; Billups et al., 2005; Jones et al., 2008). This possibility could be tested pharmacologically in future work. It is also possible that a different stimulus protocol might unmask other forms of short-term plasticity that do depend on GABA_B_ receptors, and this would need to be explored in future experiments.

The presence of presynaptic GABA_B_ receptors on inhibitory terminals in the PC has previously been inferred from experiments in which a slow component of the GABA_A_-mediated IPSC in layer 2 principal cells was shown to be inhibited by baclofen (Kapur et al., 1997). The paired-pulse depression exhibited by this slow component was also preferentially reduced by a GABA_B_ receptor antagonist (Kapur et al., 1997), similar to our finding (Figure 4) except that we measured the peak amplitude of the IPSC rather than a slow component. The slow GABA_A_ IPSC identified in the earlier study might arise from neurogliaform cells, which are known to generate GABA_A_ IPSCs with slow kinetics and which are reported in the neocortex and hippocampus to be inhibited following activation of presynaptic GABA_B_ autoreceptors (Tamás et al., 2003; Price et al., 2008; Oláh et al., 2009). We have previously shown that neurogliaform cells are common in the PC and profusely innervate SL and SP cells (Suzuki and Bekkers, 2012). It is possible that, under our stimulation conditions, the GABA_A_ IPSCs comprised a larger input from neurogliaform cells than was the case in the earlier study (Kapur et al., 1997), allowing us to observe GABA_B_ receptor-mediated modulation of the peak response rather than just a slow component. Our observation that this modulation is more prominent in SP cells, and may be absent in SL cells (Figure 4), is surprising. Future experiments would need to confirm and extend this finding. It should be noted that SL and SP cells do differ in other ways with regard to their synaptic properties (Suzuki and Bekkers, 2006, 2011), so it is plausible that more prominent GABA_B_ modulation of GABA_A_ IPSCs in SP cells is another point of difference.

The slow shift from suppression to excitation we observed when locally applying baclofen *in vivo* (Figure 5) was surprising to us. GABA_B_ receptor activation is often associated with anticonvulsant action; indeed, the GABA_B1_ knockout mouse exhibits spontaneous seizures (Schuler et al., 2001). However, baclofen has also been reported to induce seizures in patients (Schuele et al., 2005) and in animal models (Dugladze et al., 2013), evidently in a concentration-dependent manner. Thus, the role of baclofen and GABA_B_ receptors in hyperexcitability remains uncertain (Bettler et al., 2004).

Our current working hypothesis is that the biphasic effect of baclofen we observe is related to layer-specific modulation as the baclofen diffuses in from the surface. The most superficial layer, layer 1a, contains the dendrites of SL and SP cells that express postsynaptic GABA_B_ receptors (Figure 2B), so baclofen in layer 1a will hyperpolarize these cells, presumably contributing to the early suppression of spontaneous action potentials (Figure 5B). Layer 1a also contains the input fibers from the OB, but presynaptic terminals on these fibers do not express GABA_B_ receptors (Tang and Hasselmo, 1994; Franks and Isaacson, 2005; Suzuki and Bekkers, 2011). However, as baclofen diffuses down to the next layer, layer 1b, it encounters associational fibers that do express presynaptic GABA_B_ receptors (Tang and Hasselmo, 1994; Franks and Isaacson, 2005; Suzuki and Bekkers, 2011). Baclofen-mediated suppression of glutamate release from associational terminals will inhibit intracortical recurrent excitation (Poo and Isaacson, 2011), further suppressing spontaneous activity in layer 2 principal cells (Figure 5B). As it diffuses deeper, baclofen will continue to inhibit associational fibers in layers 2 and 3. However, the baclofen also increasingly encounters the GABAergic terminals of deeper interneurons, notably fast-spiking basket cells which potently inhibit the perisomatic region of SL and SP cells (Suzuki and Bekkers, 2010a, b). Assuming that the terminals of fast-spiking cells in the PC express GABA_B_ receptors, activation of these receptors will suppress feedback inhibition of SL and SP cells and have a disinhibitory effect, unleashing epileptiform activity (Figures 5E–G). Such a consequence is consistent with the fact that the PC is one of the most epileptogenic regions in the brain, on a par with the hippocampus (Vaughan and Jackson, 2014; Vismer et al., 2015). Of course, it remains possible that there are other explanations for this biphasic effect of baclofen *in vivo*, and these would need to be examined in future work.

Baclofen has been used to selectively silence associational connections in the PC *in vivo* in order to study the importance of these connections for odor processing (Poo and Isaacson, 2011). Epileptic activity was not mentioned in that report. Care must be taken when interpreting such data, given that both excitatory and inhibitory synapses are likely to be perturbed by baclofen.

What might be the functional roles of GABA_B_ receptors in the PC? Blockade of these receptors has been shown to have consequences for odor discrimination in the PC of rodents (Poo and Isaacson, 2011) and in the homologous region in insects (Riffell et al., 2014). It has also been reported that GABA_B_ receptor-mediated presynaptic inhibition at excitatory synapses in the rat PC is enhanced after the animals learn a complex olfactory-discrimination task (Kfir et al., 2014). It was proposed that this enhancement might be a homeostatic adaptation to prevent uncontrolled activity after learning-induced increases in excitability (Kfir et al., 2014). Given the diverse effects of GABA_B_ receptor activation in neural circuits (Bettler et al., 2004), it is likely that additional roles of these receptors in olfaction remain to be discovered.

In conclusion, by using a combination of *in vivo* and *in vitro* approaches, we have provided further evidence that GABA_B_ receptors are key players in the maintenance of balanced circuit behavior in the PC. By potently regulating membrane potential and neurotransmitter release, GABA_B_ receptors can function as variable modulators of complex neural processing.

## Author Contributions

LBG and MLST are joint first authors on the article and performed the experiments. MLST and JMB conceived the project and designed the experiments. LBG, MLST and JMB did the analysis and wrote the manuscript.

## Conflict of Interest Statement

The authors declare that the research was conducted in the absence of any commercial or financial relationships that could be construed as a potential conflict of interest.

## Footnotes

^1^https://imagej.nih.gov/ij/

^2^https://www.r-project.org/
